# Lymphocyte profile and cytokine mRNA expression in peripheral blood mononuclear cells of patients with recurrent respiratory papillomatosis suggest dysregulated cytokine mRNA response and impaired cytotoxic capacity

**DOI:** 10.1002/iid3.188

**Published:** 2017-08-14

**Authors:** Anna Holm, Olga Nagaeva, Ivan Nagaev, Christos Loizou, Göran Laurell, Lucia Mincheva‐Nilsson, Karin Nylander, Katarina Olofsson

**Affiliations:** ^1^ Department of Clinical Sciences Division of Otorhinolaryngology Umeå University Umeå Sweden; ^2^ Department of Clinical Microbiology Division of Clinical Immunology Umeå University Umeå Sweden; ^3^ Department of Surgical Sciences Division of Otorhinolaryngology Uppsala University Uppsala Sweden; ^4^ Department of Medical Bioscience Division of Pathology Umeå University Umeå Sweden

**Keywords:** Human, natural killer T cells, T Cells, viral/retroviral

## Abstract

**Objectives:**

Recurrent respiratory papillomatosis (RRP) is a relatively rare, chronic disease caused by Human Papilloma Virus (HPV) 6 and 11, and characterized by wart‐like lesions in the airway affecting voice and respiratory function. The majority of HPV infections are asymptomatic and resolve spontaneously, however, some individuals are afflicted with persistent HPV infections. Failure to eliminate HPV 6 and 11 due to a defect immune responsiveness to these specific genotypes is proposed to play a major role in the development of RRP.

**Methods:**

We performed a phenotypic characterization of peripheral blood mononuclear cells (PBMC) collected from 16 RRP patients and 12 age‐matched healthy controls, using immunoflow cytometry, and monoclonal antibodies against differentiation and activation markers. The cytokine mRNA profile of monocytes, T helper‐, T cytotoxic‐, and NK cells was assessed using RT‐qPCR cytokine analysis, differentiating between Th1‐, Th2‐, Th3/regulatory‐, and inflammatory immune responses.

**Results:**

We found a dominance of cytotoxic T cells, activated NK cells, and high numbers of stressed MIC A/B expressing lymphocytes. There was an overall suppression of cytokine mRNA production and an aberrant cytokine mRNA profile in the activated NK cells.

**Conclusion:**

These findings demonstrate an immune dysregulation with inverted CD4^+^/CD8^+^ ratio and aberrant cytokine mRNA production in RRP patients, compared to healthy controls.

## Introduction

Though human papilloma virus (HPV) infection is extremely common in humans, recurrent respiratory papillomatosis (RRP) is a relatively uncommon disease worldwide, with an incidence range between 3.62–4.3/100,000 for juveniles and 1.8–3.94/100,000 for adults 1, 2. RRP is characterized by benign, wart‐like lesions in the upper airway, and is associated with extensive morbidity due to its impact on voice and respiratory function, affecting quality of life 3.

Close to 200 HPV genotypes have been characterized 4, of which two, HPV6 and 11, are associated with the development of RRP.

HPV is today the world's most common sexually transmitted disease 5. However, a growing body of evidence supports the possibility that non‐sexual transmission routes also could be of importance 6. The majority of HPV infections are asymptomatic and clear spontaneously 7. Why some HPV infections lead to chronic diseases requiring lifelong treatment is unclear. It is also unknown why some parts of the airway exhibit RRP lesions, while nearby airway mucosa does not, despite the presence of HPV 8. The HPV specific cell‐mediated immune system might be crucial in HPV‐related tumor development, including RRP 9.

There is compiling evidence that failure of HPV clearance and severity of RRP disease is associated with impaired/dysregulated immune response. One report shows that RRP patients develop an ineffective HPV‐specific, T‐cell immune response 10. Recently published work has further focused on the connection between HPV infections and the Th2‐like immune response, in which CD4^+^ T‐cells induce expression of immunosuppressive cytokines (including IL‐4 and IL‐10) that suppress Th1 (cytotoxic) immunity 11, 12. Patients with RRP express a parallel decrease in IFN‐γ, IL‐12, and IL‐18 13. In addition, an elevated Th2‐like cytokine response has been associated with disease severity 10. This imbalance of Th1/Th2‐like cellular response could explain why RRP patients’ immune systems fail to clear the HPV infection and are unable to prevent the reappearance of RRP. Dysfunctional natural killer (NK‐) cells are present in RRP lesions, and they are unable to destroy HPV‐infected keratinocytes 9. The expression of major histocompatibility complex (MHC) antigens seems to play a role in patients with RRP through altered function of cell‐mediated immunity 14. MHC class I chain‐related (MIC) A and B molecules, ligands of the activating NK‐cell receptor NKG2D, have been reported as upregulated in number, and to exhibit differential expression among HPV‐infected, and non‐infected cell lines 15, 16.

The aim of this study was to characterize the phenotype of the peripheral blood mononuclear cells (PBMC) in RRP patients, in comparison to healthy controls. Our study outcome shows that a majority of the RRP patients had an inverted CD4^+^/CD8^+^ ratio (<1). Findings that prompted us to analyze and compare the cytokine mRNA profile of RRP patients with normal CD4^+^/CD8^+^ ratios as a reference group (=1) to those with the lowest CD4^+^/CD8^+^ ratios seeking for explanation of disturbed immunity as a reason for RRP infection recurrence.

## Materials and Methods

### Ethical permission

The study was conducted in accordance with the Declaration of Helsinki, and was approved by the Regional Ethical Review Board in Umeå, Sweden (Approval numbers 2015‐323‐32 M [2012‐379‐31 M], 2015‐10‐19). The ethical consent included all ages. The patients and controls donated samples after informed consent.

### Study population

Blood samples were collected from 20 randomly selected RRP patients and 12 age‐, gender‐, and comorbidity‐matched controls between January 2011 and May 2013. Randomization was secured as patients were included, when they needed treatment during the data gathering period. The control group was recruited from volunteers at the department of Otorhinolaryngology, University Hospital of Umeå, providing only venous blood samples. The RRP subjects were identified from patients who were diagnosed and/or treated at the tertiary referral center at the department of Otorhinolaryngology, University Hospital of Umeå. This department provides service (inquiry and surgical treatment) to a population of approximately 900,000 residents of the region (∼4 inhabitants per square kilometer [2015]). The sample size was determined by access of regional RRP patients during the study time and no power calculation was performed.

### Exclusion criteria

After blood samples were collected, three RRP subjects were not analyzed further due to low numbers of viable PBMC and insufficient numbers to perform flow cytometry analyses. One patient was initially included but finally not analyzed due to low age, immature immune system, and absence of comparable age‐matched control.

### Inclusion criteria

The cut‐off points for juvenile onset RRP versus adult onset RRP was defined as incidence at 18 years or younger in accordance to Omland et al. 17. The indication for surgery was based on the subjective severity of symptoms and a detailed discussion with the patient or guardian. None of the eligible subjects smoked, and none of the juvenile RRP patients were premature at birth. Patients were included regardless of occurrence of allergy, gastroesophageal reflux disease (GERD), or asthma.

The detailed outline of the patient characteristics is provided in Table 1. The respiratory papilloma (glottic‐, sub‐, and supraglottic location) were addressed according to a locally modified Derkay staging assessment system. In contrast to Derkay et al. 18, the goal was not to streamline the prediction of treatment intervals based on anatomical locations or symptom score, but rather to identify and describe the RRP lesions. Therefore, Derkay scores were not calculated. Data were stored in the northern Sweden RRP database. The main indications for surgery were hoarseness and mild airway obstruction (mild stridor at activity). Surgery was performed under general anesthesia using an AcuPulse Lumenis CO2 laser. All therapeutic interventions were performed by one otolaryngologist specialized and responsible for the RRP treatment in the northern region of Sweden (Katarina Olofsson, MD, PhD).

**Table 1:** RRP patient characteristics. Localization: 1, glottic lesion; 2, supraglottic lesion; 3, subglottic lesion

			Gender			Localization		
#	HPV type	Onset	M	F	# of surgeries	1	2	3
1	HPV6	Juvenile	X		51x	X	X	X
2	HPV11	Juvenile	X		133x	X	X	X
3	HPV6	Adult	X		11x	X	X	
4	HPV6	Adult	X		9x	X		
5	HPV6	Adult	X		4 x	X		
6	HPV6	Adult	X		7 x	X		
7	HPV6	Adult	X		17x	X		
8	HPV6	Adult	X		2x	X		
9	HPV6	Adult	X		2x	X		
10	HPV6	Adult	X		9x	X		
11	HPV16	Adult	X		6x	X		
12	not typed	Pre‐adolescence 6yrs	X		10 x	X		
13	HPV6	Adult		X	13x	X	X	
14	HPV6	Adult		X	2x	X		
15	HPV6	Adult		X	6x	X		
16	HPV16	Adult		X	8x	X		

### Patient samples, monoclonal antibodies, and consumables

Venous blood samples from 16 patients with RRP, and 12 healthy age‐matched controls, were collected and analyzed. The clones and specificities of the monoclonal antibodies (mAbs) used in this study were as follows: anti‐CD45‐FITC/CD14‐PE (clone T29/33 and TUK4), anti‐CD4‐PE (clone MT310), anti‐CD8‐PE (clone DK25), anti‐CD16‐FITC (clone DJ130c), anti‐CD19‐PE (clone HD37) (DAKO Norden A/S, Glostrup, Denmark); anti‐pan‐γδ‐FITC (clone 5A6.E9) (Endogen, Thermo Fisher Scientific Inc., Rockford, IL); anti‐CD56‐PE (clone MY31), anti‐NKG2D (clone 1D11), anti‐pan MIC (clone 6D4) (BD Biosciences Pharmingen, Franklin Lakes, NJ); CD161‐PE (clone HP‐3G10) (BioLegend, London, United Kingdom). In the negative controls for indirect immunofluorescence staining, isotype‐matched anti‐mouse IgG subclass mAbs (clones DAK‐GO1 and DAK‐GO9) (DAKO Norden A/S, Glostrup, Denmark) were used, as well as secondary FITC‐conjugated rabbit anti‐goat IgG (Jackson ImmunoResearch Laboratories, Newmarket, Suffolk, United Kingdom). The following consumables for molecular work were used: RNeasy Mini kit (QIAGEN, Hilden, Germany); High‐capacity cDNA Reverse transcription kit, catalog number 4368813, Universal Master Mix, TaqMan® assays and Eukaryotic 18S rRNA endogenous control, VIC®/MGD probe, primer limited, catalog number 4319413E (all these from Life Technologies, Thermo Fisher Scientific, Waltham, MA).

### Isolation of PBMC and purification of subpopulations of PBMC

Blood was drawn in EDTA‐containing tubes and immediately processed for peripheral blood mononuclear cell (PBMC) isolation by a standard Lymphoprep® (Nycomed, Oslo, Norway) gradient centrifugation method as previously described 19, 20. A part of the freshly isolated PBMC was used directly for immunofluorescent staining with monoclonal antibodies against phenotypic and activation markers and flow cytometry.

Subpopulations of CD14^+^, CD4^+^, CD8^+^, and CD56^+^ cells were purified from the PBMC using positive selection with immunomagnetic beads (Dynabeads, Dynal Biotech, Thermo Fisher Scientific) coated with specific antibodies according to the manufacturer's instructions. The separated subpopulations were kept at −80°C in RLT lysis buffer until molecular analyses were performed.

### Immunoflow cytometry for phenotypic characterization of PBMC

One hundred thousand living cells per well, suspended in PBS containing 3% bovine calf serum and 0.05% sodium azide, were plated in U‐shaped microtiter plates. For the analysis of most surface molecules, direct immunofluorescent staining with appropriate concentration of FITC‐ and/or PE‐conjugated mAbs was performed. NKG2D receptor and MIC A/B ligands were stained with indirect immunofluorescent staining. Appropriately labeled, isotype‐matched irrelevant mAbs were used in the negative controls for nonspecific fluorescence. As secondary antibodies FITC‐conjugated goat anti‐mouse antibodies from DACO were used. Minimum of 10,000 events for each marker were collected by Accuri C6 flow cytometer (BD Biosciences, Stockholm, Sweden) and analyzed using CFlow Plus software (BD Biosciences, Sweden).

### Real‐time quantitative reverse transcription‐polymerase chain reaction (RT‐qPCR)

A cytokine gene expression analysis was performed according to the MIQE requirements 21. Cells bound to beads were dissolved in 350 μl of RLT lysis buffer (RNeasy Mini kit, QIAGEN, Hilden, Germany) and kept at −80°C until total RNA extraction was performed. The RNA yield (on average 2574 ng in a total volume of 30 μl) and purity (average A260/A280 ratio 1.6) were assessed by NanoDrop N‐1000 spectrophotometer (Thermo Fisher Scientific). Reverse transcription was done as previously described 19, 20, 22. For each sample, multiplexed detection of both the gene of interest (FAM®/MGD probe) and a reference gene (Eukaryotic 18S rRNA endogenous control, VIC®/MGD probe, primer limited, Catalog # 4319413E, Life Technologies, Thermo Fisher Scientific) was performed. Average threshold cycle values are showed in supplementary Table S1. As optimized before 23, the reference gene was used in every individual RT‐qPCR reaction for evaluation of RNA integrity, efficiency of reverse transcription, and cDNA stability. We used premade TaqMan® assays and information regarding amplicon location/length and other details are available at www.lifetechnologies.com. Official gene symbol and assay ID number are as follows: IL1 B (Hs01555410_m1), IL2 (Hs00174114_m1), IL4 (Hs00174122_m1), IL5 (Hs00174200_m1), IL6 (Hs00985639_m1), IL8 (Hs00174103_m1), IL10 (Hs00961622_m1), IL13 (Hs00174379_m1), IL15 (Hs01003716_m1), IL17A (Hs00174383_m1), IFNG (Hs00989291_m1), TNF Hs00174128_m1), LTA (Hs04188773_g1), TGFB1 (Hs99999918_m1), and CSF2 (Hs00929873_m1). The PCR reactions were run on a 7900HT instrument (Life Technologies/Applied Biosystems, Thermo Fisher Scientific) with factory default settings for 40 cycles and the results were analyzed with a relative quantification method. Each test included no template control (NTC) as a negative assay control and a template of PMA/ionomycin‐stimulated PBMC from healthy donors acted as a positive assay control.

### Statistics

Comparative Ct (ΔΔCt) method was applied for computing relative quantities (RQ) and an average RQ (aRQ) was calculated for both groups. Individual fold difference value is defined as a result of division of the abnormal CD4^+^/CD8^+^ ratio <1 group aRQ by aRQ of the reference group with normal CD4^+^/CD8^+^ > 1. Thus, standard error and standard deviation is not applicable for calculation and presentation of the results. Statistical tests were used to estimate the variance for each group. We used Student's *T* test to evaluate statistical significance. *p*‐values ≤0.05 were considered significant.

## Results

### Phenotypic characterization of the immune cell population in patients with RRP compared to healthy controls

**Figure 1:**
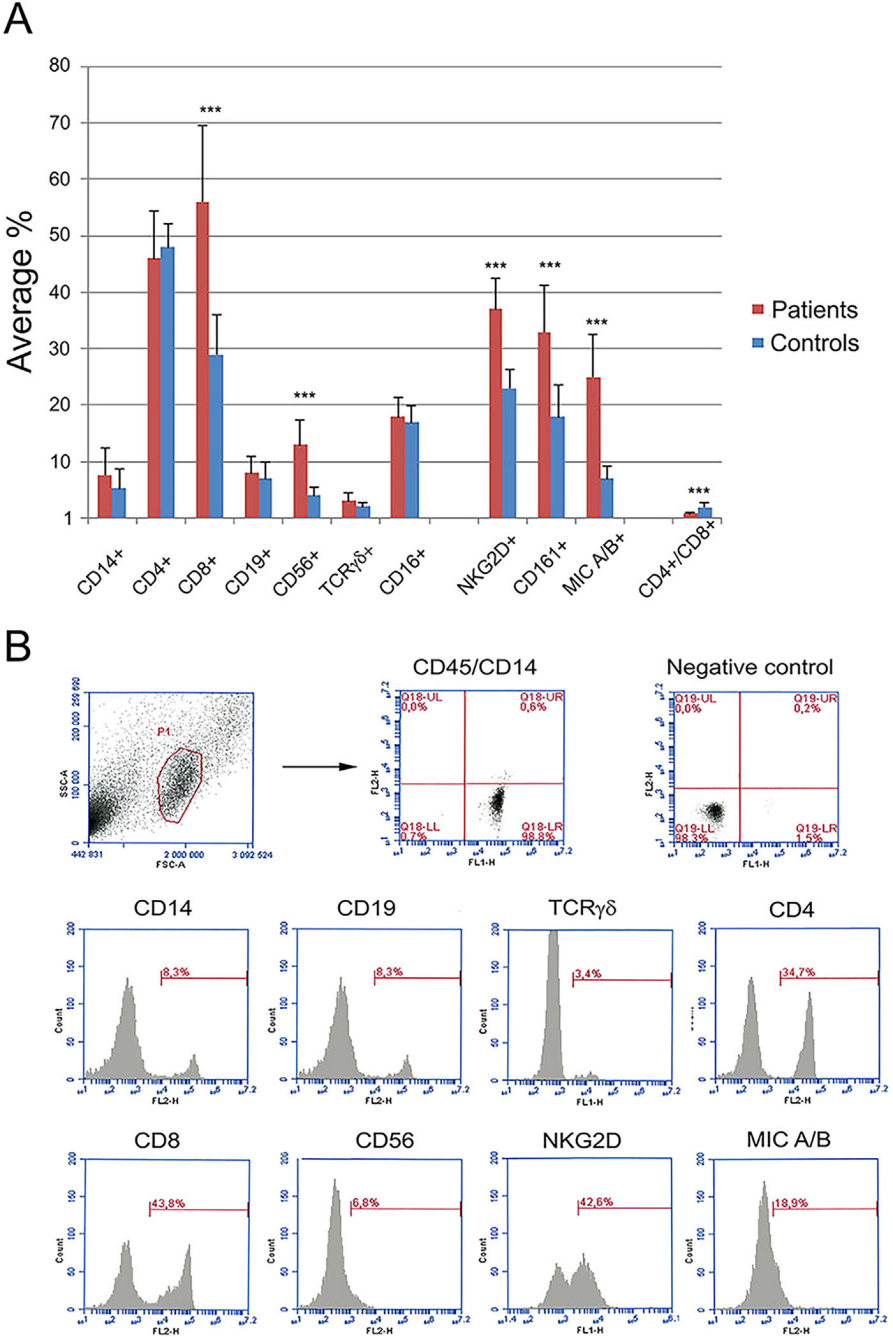
(A) Immunoflow cytometric analysis of the phenotypic lymphocyte profile and NK‐cell receptors in PBMC isolated from 16 RRP patients and 12 healthy age‐matched controls. (B) A representative immunoflow cytometric experiment showing the lymphocyte gate and histograms for the investigated lymphocyte subpopulations. Note that the average CD4^+^/CD8^+^ ratio is inverted (0.86) in RRP patients compared to controls (1.97). Statistically significant changes are designated as follows * = *p *<0.05; ** = *p *< 0.01; and *** = *p *< 0.001.

PBMC were stained for leukocyte differentiation markers in order to estimate their phenotypic profile. The expression of NK cell receptors was evaluated by analyzing the total expression of the NK‐cell receptors CD161, NKG2D, and the NKG2D ligands MIC A and B. The results are summarized and presented in Figure 1. There was a higher proportion of CD8^+^ T cytotoxic/suppressor cells (*p *< 0.001) and CD56^+^ NK‐cells (*p *< 0.001) in the peripheral blood of RRP patients compared to healthy controls (Fig. 1 and Table 2). In contrast, no changes were observed in the proportion of CD4^+^ T helper (Th) cells, B cells, monocytes, and γδ‐T cells. In addition, there was a higher expression of the NKG2D receptor (*p* < 0.001) and a strong tendency for higher CD161 expression in RRP patients’ lymphocytes compared to healthy controls. More than 40% of the RRP lymphocytes expressed the NKG2D ligands MIC A/B, making them susceptible for NKG2D attack and destruction. Notably, the ratio of CD4^+^/CD8^+^ cells was converted from the normal value >1–<1. Twelve out of 16 RRP patients (75%) had a CD4^+^/CD8^+^ ratio <1. Eight out of 16 (50%) RRP patients had CD4^+^/CD8^+^ ratio between 0.7 and 0.9. Four RRP patients (25%) had ratios of 0.45, 0.54, 0.61, and 0.64, and four patients (25%) had a normal ratio of 1.40, 1.15, 1.93, and 1.02; that is, a CD4^+^/CD8^+^ ratio >1. In summary, the phenotypic lymphocyte profile of RRP patients showed an increased percentage of cytotoxic/suppressor T‐ and NK‐cells, which was not accompanied by adequate T‐helper cell increase, resulting in an inverted CD4^+^/CD8^+^ ratio. In parallel, there was an increased expression of the activating NK‐cell receptor NKG2D and its ligands MIC A and B. The CD4^+^/CD8^+^ ratio reflects the proportion between T helper and T cytotoxic effector cells in the peripheral blood. In individuals with healthy immune systems the ratio should be above 1. An inverted CD4^+^/CD8^+^ ratio is a sign of a compromised immune system, well‐documented in HIV patients who enter the AIDS phase of the disease when the CD4^+^/CD8^+^ ratio is inverted 24. Hence, we tried to correlate clinical characteristics of RRP patients to the measured CD4^+^/CD8^+^ ratio, but such a correlation could not be found. The CD4^+^ T helper cells are instrumental for regulation of the immune responses. Therefore, since this finding was extraordinary, we wanted to compare the cytokine mRNA profiles of RRP patients with normal CD4^+^/CD8^+^ ratios (1.40, 1.15, 1.93, and 1.02) to RRP patients with the lowest CD4^+^/CD8^+^ ratios (0.45, 0.54, 0.61, and 0.64) using real‐time RT‐qPCR.

**Table 2:** Average percentage ± SD of each cell populations in patients and control groups

Lymphocytes/CD marker	Patients % positive cells ± SD	Controls % positive cells ± SD
T helper cells/CD4^+^	45.5 ± 8.3	48.0 ± 4.1
Cytotoxic T cells/CD8^+^	56.4 ± 13.6	29.4 ± 6.9
B cells/CD19^+^	8.2 ± 2.9	6.5 ± 3.0
Monocytes/CD14^+^	7.6 ± 4.8	5.3 ± 3.5
γδ cells/TCRγδ^+^	2.9 ± 1.5	1.9 ± 0.8
NK cells/CD56^+^	12.5 ± 4.3	3.6 ± 1.4

### RRP patients with the lowest inverted CD4±/CD8± ratio had a generally lower and aberrant cytokine mRNA expression compared to those with normal CD4±/CD8± ratio

**Figure 2:**
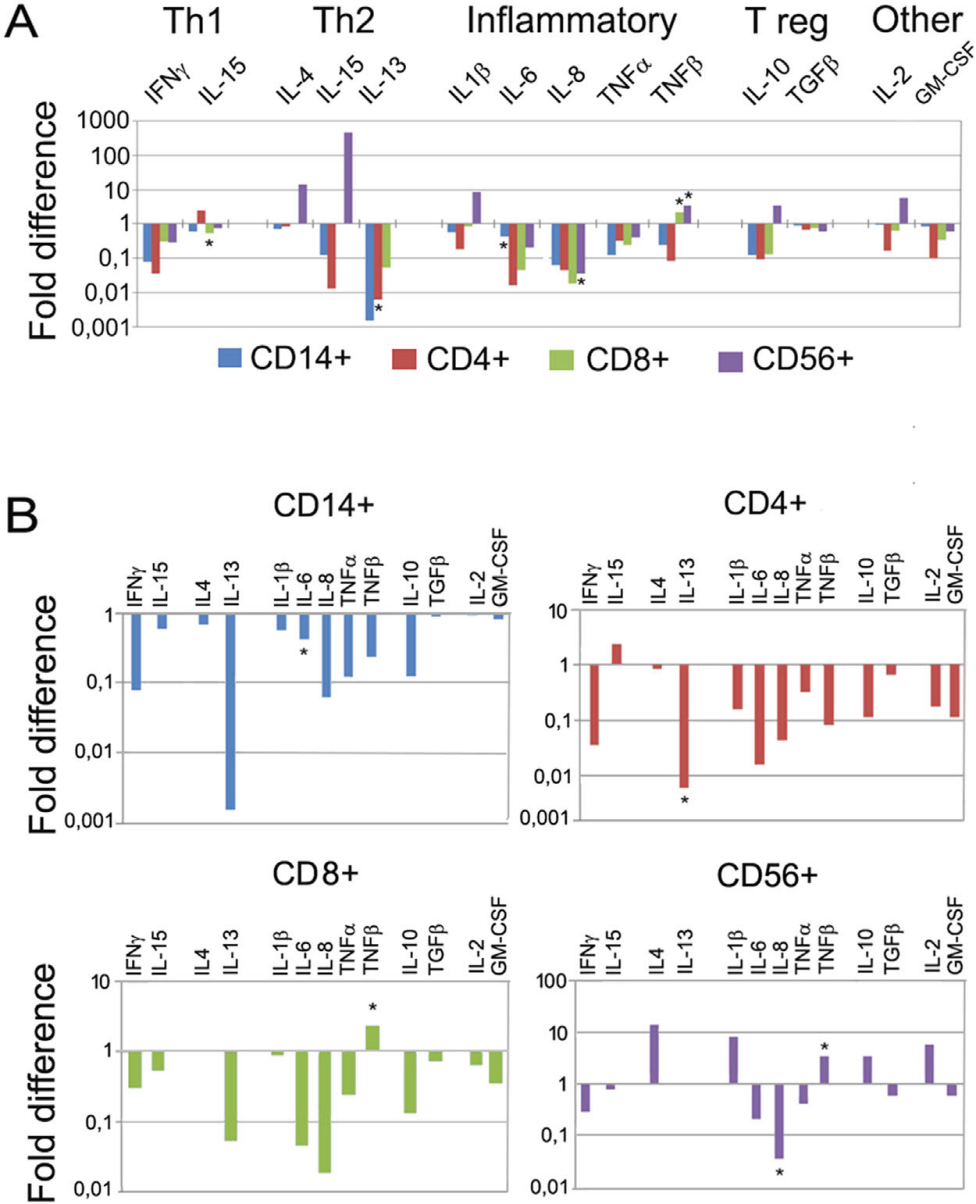
Cytokine mRNA profiles of relative mRNA expression analyzed by quantitative RT‐PCR in CD14^+^, CD4^+^, CD8^+^, and CD56^+^ cells, purified from patients with RRP. Two groups of 4 RRP patients each, those with CD4^+^/CD8^+^ ratio >1 and those with CD4^+^/CD8^+^ ratio <1, were compared. The cytokine mRNA profiles for all tested cytokines grouped into Th1, Th2, inflammatory‐, and Th3 regulatory responses are shown in (A) and the same cytokine mRNA profiles grouped for the individual lymphocyte subpopulations are shown in (B). The results are presented as fold‐difference of cytokine mRNA expression level between the group of RRP patients with inverted CD4^+^/CD8^+^ ratio <1 and the group of RRP patients with normal CD4^+^/CD8^+^ ratio >1 which represented the reference group with expression = 1. Thus, standard error and standard deviation is not applicable for calculation and presentation of the results. Note the overall suppression of cytokine mRNA response in RRP patients with inverted CD4^+^/CD8^+^ ratio and the aberrant cytokine mRNA expression in CD56^+^/NK cells. The limited number of cases included does not allow reliable statistical analysis.

In order to further address these findings, we looked for differences in cytokine mRNA expression between the patient group with highly suppressed CD4^+^/CD8^+^ ratio (<1, *n* = 4) and those with normal CD4^+^/CD8^+^ ratio (>1, *n* = 4). For each patient, the mRNA expression profiles were investigated in purified CD14^+^cells (monocytes), CD4^+^/T helper cells, CD8^+^ T cytotoxic/suppressor cells (CTL), and CD56^+^ NK cells. mRNA expression for 14 cytokines, chosen to discriminate between T helper Th1‐, Th2‐, inflammatory‐, and Th3 regulatory immune responses, was investigated using real time RT‐qPCR and specific primers and probes. The relative cytokine expression between the two groups was calculated and compared, setting the cytokine expression in immune cells of patients with “normal” CD4^+^/CD8^+^ ratio of >1 as a reference = 1. The results are summarized in Figure 2A and B. In Figure 2A, the fold differences of the relative expression of 14 cytokines, expressed by four cell types are shown. Patients with CD4^+^/CD8^+^ ratio >1 are referred to as the reference group = 1. The cytokine mRNA expressions in patients with CD4^+^/CD8^+^ ratio <1 are shown as histogram staples calculated in comparison to the reference group. There was a general downregulation of the cytotoxic response in patients with inverted CD4^+^/CD8^+^ ratio. Noteworthy, the cytokines of crucial importance for clearing a viral infection, such as the Th1 cytokines IFNγ, and IL‐15, the pro‐inflammatory cytokines IL‐1β, IL‐6, TNFα, and TNFβ/lymphotoxin, were downregulated. The lymphotropic cytokine IL‐2 was suppressed in all lymphocyte subpopulations but one (Fig. 2B). In Figure 2B, the same relative cytokine mRNA expression is shown, grouped in individual figures for each tested lymphocyte subpopulation. Interestingly, there was an abnormal cytokine mRNA response in the CD56^+^ NK‐cells (Fig. 2B). These NK‐cells were the only cell types that upregulated IL‐2 expression consistent with cell activation and clonal expansion. However, their overall cytokine mRNA response is characterized by (i) inhibition of the typical NK‐cell cytokines like IFNγ, IL‐15, TNFα, and β that promote Th1 response; (ii) aberrant expression and up‐regulation of IL‐4, IL‐5, that is, switching the cytokine profile from cytotoxic/anti‐viral/Th1 to humoral Th2 response; and (iii) up‐regulation of IL‐10, a regulatory cytokine that promotes adaptive peripheral Tr1 response, and immune suppression. Furthermore, the cytokine mRNA production by CD8^+^T cells that should be responding with a cytotoxic response is entirely suppressed. Similarly, there was a cytokine mRNA suppression in the CD4^+^T helper cells.

## Discussion

The aim of the current study was to characterize the phenotype of peripheral blood lymphocytes in 16 RRP patients in comparison to 12 healthy age‐ and gender‐matched controls using immunoflow cytometry. In addition, the cytokine mRNA profile was investigated for a panel of cytokines distinguishing between Th1‐, Th2‐, Th3/Tr1 regulatory, and inflammatory responses.

Our results can be summarized as follows: (i) There was a statistically significant upregulation of the percentage of cytotoxic T cells in comparison to T helper cells in the peripheral blood of patients compared to controls resulting in an inverted CD4^+^/CD8^+^ ratio (<1) in the majority of the RRP patients. (ii) The NK‐cell population in RRP patients was characterized by significantly elevated expression of the NK‐cell receptors NKG2D and CD161 compared to normal controls. (iii) A large proportion of PBMC in RRP patients expressed the stress‐related NKG2D ligands MIC A/B, making these cells potential targets for NK‐cell attack. (iv) The cytokine mRNA profile in RRP patients with the lowest CD4^+^/CD8^+^ ratio was generally suppressed in monocytes, T helper cells, cytotoxic T cells, and NK cells compared to patient with normal CD4^+^/CD8^+^ ratio >1. In addition, the cytokine mRNAs expressed by the NK‐cells had a dysregulated profile with downregulated IFNγ mRNA expression, and upregulated immunosuppressive cytokines characteristic for regulatory response.

An interesting observation was that the ratio of CD4^+^/CD8^+^ in the peripheral blood of 12/16 RRP patients was inverted from the expected normal value >1–<1, a value commonly observed in HIV patients entering the AIDS phase of the disease 24. In contrast, an earlier study from 1994 14 did not detect any differences in T cell subsets, when comparing RRP patients to healthy individuals, however, they assumed that T suppressor cells could locally be involved in RRP. The diverging outcomes could be the result of differences in methods and study populations. At present, the reasons behind the inverted CD4^+^/CD8^+^ ratio in RRP are not clear. However, one has to keep in mind that sample size was small in general and in particular in the subgroup with CD4^+^/CD8^+^ <1. A possible cause for the inverted CD4^+^/CD8^+^ ratio in RRP patients might be that patients had acquired their disease a long time before the single phenotype analysis was performed. It would have been desirable to perform longitudinal flow cytometric analyses at different time points in order to address the phenotypic changes over time and thus possibly find an explanation for the inverted CD4^+^/CD8^+^ ratio. However, such a study would neither be possible nor ethically justified since part of the patient material has been vaccinated against HPV with an expected change in the phenotype of the lymphocytes.

The cytokine mRNA profile investigation of RRP patients showed profoundly depressed cytokine mRNA production in all tested cytokines by CD8^+^ cells, implying a suppressed cytotoxic function. The mRNA cytokine expression in CD4^+^ cells was downregulated as well, impairing their helper cell function.

Similar to CD8^+^ cells, and in accordance with earlier studies 25, there was an increased percentage of CD56^+^ NK‐cells, combined with an enhanced expression of the activating NK cell‐receptors NKG2D, CD161, and NKG2D ligands MIC A/B in the RRP patients. NKG2D and CD161 receptors function as main receptors in NK cells and as co‐receptors in CTLs 26, 27. Here, we report a threefold increase of PBMCs expressing MIC A/B in the peripheral blood of RRP patients, compared to healthy controls. MIC A/B molecules serve as molecules tagging DNA damaged cells and are expressed in various conditions of biological stress, such as malignant tumors 28, viral and bacterial infections 29, 30, and autoimmune conditions 31. MIC A/B are ligands of the NKG2D receptor 32 responsible for elimination of stressed cells, serving as a major immune surveillance receptor 33, 34. It has been shown that infections 29, celiac disease 35, and malignant tumors 28 can upregulate MIC A/B expression on lymphocytes dysregulating the NK‐cell response. The high percentage of PBMC expressing MIC A/B found in RRP patients suggests that they could be targets for NK‐cell attack. Overall, these findings support the idea that a dysregulated antiviral response is a possible reason for persistent infection in RRP patients. At present, we do not know the phenotype of the lymphocytes expressing MIC A/B. Our findings of the downregulated cytokine mRNA expression in the RRP patient group with the lowest CD4^+^/CD8^+^ ratio might indicate an elimination of CD4^+^ cells expressing MIC A/B since the percentage of these lymphocytes did not parallel the elevation of the CD8^+^CTL. More studies are needed to address the origin of the elevated numbers of PBMCs expressing MIC A/B. Other issues to address are the cytotoxic capacity of CD8^+^ T‐ and NK‐cells in RRP patients compared to normal controls, as well as HPV‐induced toxicity for selective lymphocyte populations in RRP patients.

In conclusion, despite the limited number of samples, our study shows that patients with RRP have an immune dysregulation with inverted CD4^+^/CD8^+^ ratio, profoundly downregulated T helper cell response, and aberrant cytokine mRNA production by CD56^+^ NK‐cells.

Further studies are needed to elucidate the mechanism(s) behind these observations. The results from this report need to be interpreted with caution, due to small sample size, and moderate statistical power which might affect the reliability of the study outcome.

## Authors’ Contributions

K. Olofsson was in charge of the patients and collected the samples; O. Nagaeva and I. Nagaev performed the experiments; K. Olofsson, L. Mincheva‐Nilsson, C. Loizou, G. Laurell, and K. Nylander designed the study; A. Holm, L. Mincheva‐Nilsson, C. Loizou, K. Nylander, G. Laurell, O. Nagaeva, I. Nagaev, and K. Olofsson wrote the paper. Michael Haney revised the text.

## Conflict of Interest

None declared.

## Supporting information

Additional supporting information may be found in the online version of this article at the publisher's web‐site.

**Table S1**. Average threshold cycle values for FAM/VIC for each test subgroup: For each sample, multiplexed detection of both the gene of interest (FAM®/MGD probe) and a reference gene (Eukaryotic 18S rRNA endogenous control, VIC®/MGD probe, primer limited, Catalog # 4319413E, Life Technologies, Thermo Fisher Scientific, USA) was performed and average Ct (threshold cycle) values are shown here.

**Table S2**. Fold‐difference of cytokine mRNA expression level between the group of RRP patients with inverted CD4+/CD8+ratio <1 and the reference group of RRP patients with normal CD4/CD8 ratio >1 (expression level =1).
