# Supplementary material for: Lymphocyte profile and cytokine mRNA expression in peripheral blood mononuclear cells of patients with recurrent respiratory papillomatosis suggest dysregulated cytokine mRNA response and impaired cytotoxic capacity
Source: Immun Inflamm Dis. 2017 Aug 14;5(4):541–50. doi: 10.1002/iid3.188 (PMC5691300; doi:10.1002/iid3.188)
Supplement: Supplementary file 1 — Table S1. Average threshold cycle values for FAM/VIC for each test subgroup: For each sample, multiplexed detection of both the gene of interest (FAM®/MGD probe) and a reference gene (Eukaryotic 18S rRNA endogenous control, VIC®/MGD probe, primer limited, Catalog # 4319413E, Life Technologies, Thermo Fisher Scientific, USA) was performed and average Ct (threshold cycle) values are shown here. [file IID3-5-541-s001.doc]

|  | | | |  |  | |  |  | |  |  | |  |
| --- | --- | --- | --- | --- | --- | --- | --- | --- | --- | --- | --- | --- | --- |
|  |  | CD14^+^ | | | CD4^+^ | | | CD8^+^ | | | CD56^+^ | | |
|  |  | FAM | VIC | | FAM | VIC | | FAM | VIC | | FAM | VIC | |
| IFNγ | Inverted ratio | 31,23 | 14,21 | | 32,72 | 14,23 | | 30,46 | 13,78 | | 31,27 | 15,49 | |
|  | Normal Ratio | 30,33 | 14,20 | | 28,35 | 14,35 | | 29,14 | 13,81 | | 28,56 | 13,30 | |
| IL-15 | Inverted ratio | 29,19 | 13,76 | | 34,68 | 16,69 | | 32,94 | 13,54 | | 32,36 | 14,69 | |
|  | Normal Ratio | 30,18 | 13,56 | | 32,72 | 14,12 | | 31,94 | 13,68 | | 30,41 | 13,35 | |
|  |  |  |  | |  |  | |  |  | |  |  | |
| IL-4 | Inverted ratio | 36,47 | 13,91 | | 36,95 | 17,16 | | ND | 13,64 | | 36,79 | 17,08 | |
|  | Normal Ratio | 37,08 | 13,95 | | 37,64 | 14,52 | | 38,06 | 13,93 | | 36,80 | 13,49 | |
| IL-13 | Inverted ratio | 39,69 | 14,39 | | 36,77 | 17,19 | | 38,77 | 14,07 | | 29,25 | 17,38 | |
|  | Normal Ratio | 35,11 | 14,59 | | 32,25 | 14,60 | | 37,14 | 14,20 | | 39,74 | 14,22 | |
|  |  |  |  | |  |  | |  |  | |  |  | |
| IL-1β | Inverted ratio | 27,10 | 13,69 | | 26,87 | 14,65 | | 32,53 | 13,77 | | 32,33 | 15,06 | |
|  | Normal Ratio | 27,70 | 14,33 | | 33,25 | 14,31 | | 31,97 | 13,77 | | 36,12 | 14,18 | |
| IL-6 | Inverted ratio | 36,24 | 14,04 | | 37,52 | 16,77 | | 37,74 | 13,67 | | 34,17 | 14,65 | |
|  | Normal Ratio | 35,76 | 14,18 | | 33,01 | 14,29 | | 35,80 | 13,60 | | 33,05 | 13,59 | |
| IL-8 | Inverted ratio | 29,23 | 13,94 | | 32,47 | 16,87 | | 34,71 | 13,96 | | 33,30 | 14,81 | |
|  | Normal Ratio | 24,24 | 13,87 | | 30,93 | 14,63 | | 28,82 | 13,67 | | 27,28 | 12,91 | |
| TNFα | Inverted ratio | 29,30 | 14,18 | | 31,10 | 16,59 | | 31,06 | 13,46 | | 30,59 | 14,73 | |
|  | Normal Ratio | 28,12 | 14,03 | | 28,23 | 14,25 | | 29,60 | 13,72 | | 27,87 | 13,51 | |
| TNFβ | Inverted ratio | 30,25 | 13,87 | | 29,79 | 16,72 | | 28,11 | 13,47 | | 28,08 | 14,83 | |
|  | Normal Ratio | 29,82 | 13,70 | | 27,18 | 14,63 | | 29,19 | 13,31 | | 29,35 | 13,12 | |
|  |  |  |  | |  |  | |  |  | |  |  | |
| IL-10 | Inverted ratio | 35,29 | 13,93 | | 37,60 | 15,70 | | 37,93 | 13,06 | | 36,55 | 16,66 | |
|  | Normal Ratio | 32,71 | 13,25 | | 34,48 | 13,49 | | 36,31 | 13,49 | | 36,09 | 13,10 | |
| TGFβ | Inverted ratio | 25,49 | 13,48 | | 25,13 | 13,84 | | 25,65 | 14,01 | | 24,76 | 14,42 | |
|  | Normal Ratio | 22,95 | 11,50 | | 23,94 | 12,26 | | 23,98 | 12,71 | | 24,40 | 12,94 | |
|  |  |  |  | |  |  | |  |  | |  |  | |
| IL-2 | Inverted ratio | 37,27 | 14,99 | | 37,55 | 17,63 | | 36,16 | 14,30 | | 36,43 | 18,47 | |
|  | Normal Ratio | 37,83 | 15,40 | | 35,49 | 17,78 | | 35,62 | 13,91 | | 37,55 | 23,29 | |
| GM-CSF | Inverted ratio | 36,89 | 14,70 | | 37,83 | 16,76 | | 37,87 | 13,44 | | 33,21 | 16,80 | |
|  | Normal Ratio | 33,42 | 14,35 | | 33,41 | 14,42 | | 36,56 | 13,70 | | 35,96 | 13,74 | |

Supplementary table S1: Average threshold cycle values for FAM/VIC for each test subgroup: For each sample, multiplexed detection of both the gene of interest (FAM®/MGD probe) and a reference gene (Eukaryotic 18S rRNA endogenous control, VIC®/MGD probe, primer limited, Catalog # 4319413E, Life Technologies, Thermo Fisher Scientific, USA) was performed and average Ct (threshold cycle) values are shown here.
