# Supplementary material for: Lymphocyte profile and cytokine mRNA expression in peripheral blood mononuclear cells of patients with recurrent respiratory papillomatosis suggest dysregulated cytokine mRNA response and impaired cytotoxic capacity
Source: Immun Inflamm Dis. 2017 Aug 14;5(4):541–50. doi: 10.1002/iid3.188 (PMC5691300; doi:10.1002/iid3.188)
Supplement: Supplementary file 2 — Table S2. Fold‐difference of cytokine mRNA expression level between the group of RRP patients with inverted CD4+/CD8+ratio <1 and the reference group of RRP patients with normal CD4/CD8 ratio >1 (expression level =1). [file IID3-5-541-s002.doc]

|  | Th1 | | Th2 | | | Inflammatory | | | | | T regulatory | | Other | |
| --- | --- | --- | --- | --- | --- | --- | --- | --- | --- | --- | --- | --- | --- | --- |
|  | INF-γ | IL-15 | IL-4 | IL-5 | IL-13 | IL-1β | IL-6 | IL-8 | TNF-α | TNF-β | IL-10 | TGF-1β | IL-2 | GMCSF |
| CD14^+^ | 0,0787 | 0,6123 | 0,7060 | 0,1242 | 0,0015 | 0,5805 | 0,4254* | 0,0616 | 0,1214 | 0,2387 | 0,1251 | 0,9154 | 0,9754 | 0,8516 |
| CD4^+^ | 0,0364 | 2,4018 | 0,8495 | 0,0127 | 0,0062* | 0,1822 | 0,0160 | 0,0437 | 0,3296 | 0,0851 | 0,0954 | 0,6781 | 0,1591 | 0,0988 |
| CD8^+^ | 0,3007 | 0,5275* | na # | na | 0,0527 | 0,8522 | 0,0448 | 0,0181 | 0,2365 | 2,2510* | 0,1335 | 0,7373 | 0,6374 | 0,3423 |
| CD56^+^ | 0,2895 | 0,7684 | 14,0518 | 487,4981 | na | 8,5103 | 0,2027 | 0,0357* | 0,4006 | 3,4248* | 3,4620 | 0,5903 | 5,7685 | 0,5943 |

Supplementary table S2: Fold-difference of cytokine mRNA expression level between the group of RRP patients with inverted CD4^+^/CD8^+^ ratio <1 and the reference group of RRP patients with normal CD4/CD8 ratio >1 (expression level =1).

* p-value <0.05. # na, not available since mRNA was not detected.
